# Biomechanical comparison of PFNA combined with the modified candy-package wiring technique versus PFNA alone in complex unstable intertrochanteric femoral fractures: A biomechanical model study

**DOI:** 10.1097/MD.0000000000050596

**Published:** 2026-09-11

**Authors:** Qiong Chen, Xiaan Chen, Suqi Wu, Hansong Wu, Rongrong Chi, Xiangu Li, Jiaqi Wang

**Affiliations:** a Jinhua City Traditional Chinese Medicine Hospital, Jinhua City, Zhejiang Province, China; b Canwell Medical Co., Ltd., Jinhua City, Zhejiang Province, China; c School of Clinical Medicine, Hangzhou Medical College, Hangzhou City, Zhejiang Province, China.

**Keywords:** axial compression, biomechanical model, intertrochanteric femoral fracture, modified candy-package wiring technique, proximal femoral nail antirotation, rotational control

## Abstract

Proximal femoral nail antirotation (PFNA) may not provide sufficient control of unstable fracture fragments in complex intertrochanteric fractures. The modified candy-package wiring technique may supplement PFNA by improving resistance to rotational and shear displacement. This biomechanical model study compared PFNA combined with modified candy-package wiring with PFNA alone in an Evans type III intertrochanteric fracture model. Ten standardized femoral fracture models were randomly allocated to a PFNA group (n = 5) or a PFNA plus modified candy-package wiring group (PFNA + MCW group; n = 5). Torsional and axial compression tests were performed. Three repeated loading cycles were conducted for each specimen as conditioning cycles; the prespecified third cycle was used for analysis. Torsional stiffness was calculated as the change in torque divided by the corresponding change in angular displacement (Kt = Δ*T*/Δ*θ*). The mean torsional angle was significantly lower in the PFNA + MCW group than in the PFNA group (2.09 ± 0.24° vs 2.90 ± 0.18°; *P* = .0007). Torsional stiffness was also significantly lower in the PFNA + MCW group than in the PFNA group (0.48 ± 0.0529 vs 0.59 ± 0.0850 N·m/°; *P* = .045). The maximum failure load was numerically higher in the PFNA + MCW group than in the PFNA group (571.26 ± 92.75 N vs 510.34 ± 76.59 N), although the difference was not statistically significant (*P* = .291). In this small biomechanical model study, PFNA combined with modified candy-package wiring reduced torsional displacement and showed favorable trends in axial load-bearing performance compared with PFNA alone. Because several outcomes were not statistically significant and only 5 models were included per group, these findings should be interpreted as preliminary biomechanical evidence rather than proof of superiority. Larger biomechanical studies and clinical validation are required.

## 1. Introduction

Intertrochanteric femoral fractures, which occur between the greater and lesser trochanters of the femur, are common in elderly individuals, particularly those with osteoporosis.^[1–4]^ These fractures are typically caused by low-energy trauma, such as falls, in older adults, but may result from high-energy trauma in younger patients. The Evans classification system is commonly used to describe different types of intertrochanteric fractures, with Evans type III fractures being categorized as unstable. These fractures are characterized by significant displacement of fracture fragments, resulting in severely compromised stability in the fracture region.^[5]^ Treating such fractures presents considerable challenges, as the instability of fracture fragments can lead to fixation failure or delayed union.^[6,7]^

Evans type III intertrochanteric fractures generally require surgical intervention. Common surgical techniques include the use of dynamic hip screws (DHS) or proximal femoral nails to stabilize the fracture, restore lower limb function, and reduce the risk of complications associated with prolonged bed rest.^[8,9]^ While DHS has been widely applied in clinical practice, it has notable limitations. For instance, DHS provides suboptimal fixation in unstable fractures, restricts postoperative weight-bearing, and may result in complications such as femoral head collapse due to sliding.^[10]^ Although proximal femoral nails offers better axial stability, it poses technical challenges during surgery and has limited rotational control of fracture fragments, increasing the risk of complications.^[11]^ To address these challenges, new surgical strategies are required to improve multi-plane fixation stability, minimize surgical trauma, allow early weight-bearing, and provide individualized fixation solutions to enhance treatment efficacy and reduce complications.

Proximal femoral nail antirotation (PFNA) is an intramedullary fixation device commonly used to treat intertrochanteric fractures. PFNA provides excellent axial stability and anti-rotation capabilities through its proximal intramedullary design.^[12,13]^ However, when used alone in complex or comminuted intertrochanteric fractures with multiple unstable fragments, PFNA may fail to fully control the rotation and displacement of all fracture fragments. This limitation could negatively impact fracture stability and healing outcomes. To address these issues, PFNA combined with the modified candy-package wiring technique has been proposed and widely applied. This technique involves using the modified candy-package wiring to encircle and secure the greater trochanter, lesser trochanter, and other fracture fragments, in addition to PFNA fixation. This approach enhances multi-plane stability by bundling the fracture fragments, resembling the wrapping of a candy.^[14]^ Specifically, the cerclage technique supplements PFNA’s shortcomings by externally stabilizing fracture fragments, effectively preventing their rotation and displacement, and thereby significantly improving overall fracture stability.

Studies have shown that PFNA combined with the modified candy-package wiring technique offers several advantages. This approach enhances fixation stability, particularly in the management of complex and comminuted fractures, and effectively reduces secondary displacement of fracture fragments. It facilitates early fracture healing and promotes postoperative functional recovery. With improved overall fixation stability, patients can commence early weight-bearing and functional exercises, reducing complications associated with prolonged immobilization, such as deep vein thrombosis and pulmonary infections. This combined technique not only improves fracture treatment outcomes but also provides better postoperative rehabilitation opportunities for patients, demonstrating significant clinical application value.

Therefore, this study aims to construct an Evans type III intertrochanteric fracture model and conduct biomechanical tests to compare the stability and anti-displacement capabilities of PFNA combined with the modified candy-package wiring technique versus PFNA alone. The findings are intended to guide clinical treatment and postoperative rehabilitation strategies, optimize surgical techniques, and ultimately improve patients’ recovery quality and fracture healing success rates.

## 2. Materials and methods

### 2.1. Construction of Evans type III intertrochanteric femoral fracture model

A standardized Evans type III intertrochanteric femoral fracture model was created by performing an intertrochanteric osteotomy and a second osteotomy parallel to the intertrochanteric line at the upper margin of the lesser trochanter, thereby creating a free lesser-trochanter fragment. Ten simulated femoral models were standardized to a length of 25 cm and randomly allocated to 2 fixation groups (n = 5 per group). In the PFNA group, fixation was performed using PFNA alone, and the lesser-trochanter fragment was left without supplementary fixation.^[15]^ In the PFNA + MCW group,^[16]^ the same PFNA construct was supplemented with the modified candy-package wiring technique to secure the lesser-trochanter fragment. Because only non-biological simulated femoral models were used and no human participants, identifiable human tissue, or live animals were involved, institutional ethics approval and informed consent were not required.

### 2.2. Torsion testing

A preload of 2 N·m was applied for 30 minutes to reduce creep-related effects, after which the system was zeroed. Torque was then increased gradually while torque and angular displacement were recorded. Each specimen underwent 3 repeated loading cycles under the same protocol. The first 2 cycles were treated as conditioning cycles, and the prespecified third cycle was used for the statistical analysis to minimize viscoelastic and settling effects. Torque values at angular displacements of 0°, 0.5°, 1.0°, 1.5°, 2.0°, 2.5°, 3.0°, and 3.5° were extracted from the torque-angle curve. Torsional stiffness (Kt) was defined as the slope of the linear region of the torque-angle curve and calculated as Kt = Δ*T*/Δθ, where Δ*T* is the change in torque and Δθ is the corresponding change in angular displacement. The coefficient of determination (*R*^2^) from the linear regression was used to evaluate the goodness of fit of the selected linear region.^[17]^

### 2.3. Compression testing

Compression was applied at 2 mm/min. Each specimen received 3 500-N preconditioning cycles with complete unloading between cycles. These cycles were used only for conditioning and were not analyzed as independent observations. The third load–displacement record for each specimen was used for analysis. Compressive loads of 300, 600, and 900 N were applied sequentially, and the corresponding axial displacement was recorded at each load level. Loading was subsequently continued until failure. Failure was defined as a secondary fracture, implant breakage, implant penetration into the femoral head, a neck–shaft angle < 110°, or displacement > 5 mm.

### 2.4. Statistical analysis

Statistical analyses were performed at the specimen level. Continuous variables are presented as mean ± standard deviation, with the unit shown for each outcome. Because each group contained only 5 specimens, distributions were evaluated using the Shapiro–Wilk test together with inspection of histograms and Q–Q plots; however, these diagnostics have limited power at n = 5 and were interpreted cautiously. Homogeneity of variance was examined using Levene test. Welch independent-samples *t* tests were selected as the primary between-group analysis because they do not require equal variances. Two-sided *P* values and mean differences with 95% confidence intervals were reported. Torsional stiffness for each specimen was obtained from ordinary least-squares regression of torque on angle over the prespecified linear range. Repeated conditioning cycles were not entered as separate observations. No imputation was performed because no missing values were reported. Statistical significance was defined as *P* < .05.

## 3. Results

### 3.1. Evans type III intertrochanteric femoral fracture model

**Figure 1.**
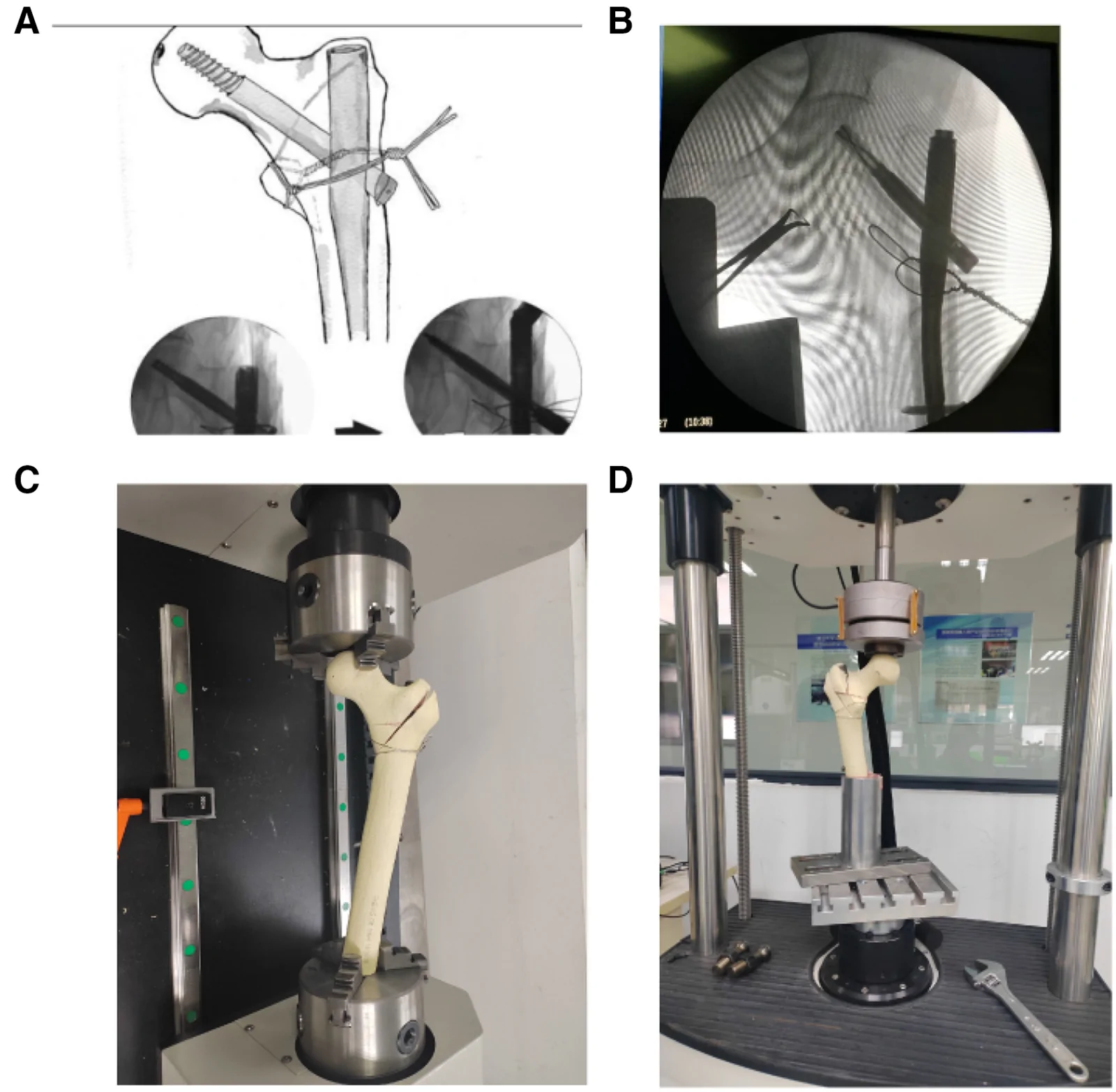
Schematic representation of the Evans type III intertrochanteric femoral fracture model construction. (A) Schematic diagram of the modified candy-package wiring technique. (B) Intraoperative visualization of the fixation outcome. (C) Torsion testing setup. (D) Compression testing setup. Note: Panels A–D show the fixation technique and the corresponding biomechanical testing configurations. MCW = modified candy-package wiring, PFNA = proximal femoral nail antirotation.

The standardized Evans type III intertrochanteric fracture models and testing configurations are shown in Figure 1. Figure 1A and 1B demonstrate the modified candy-package wiring construct, whereas Figure 1C and 1D show the torsion and compression testing setups, respectively.

### 3.2. Torsion test

**Figure 2.**
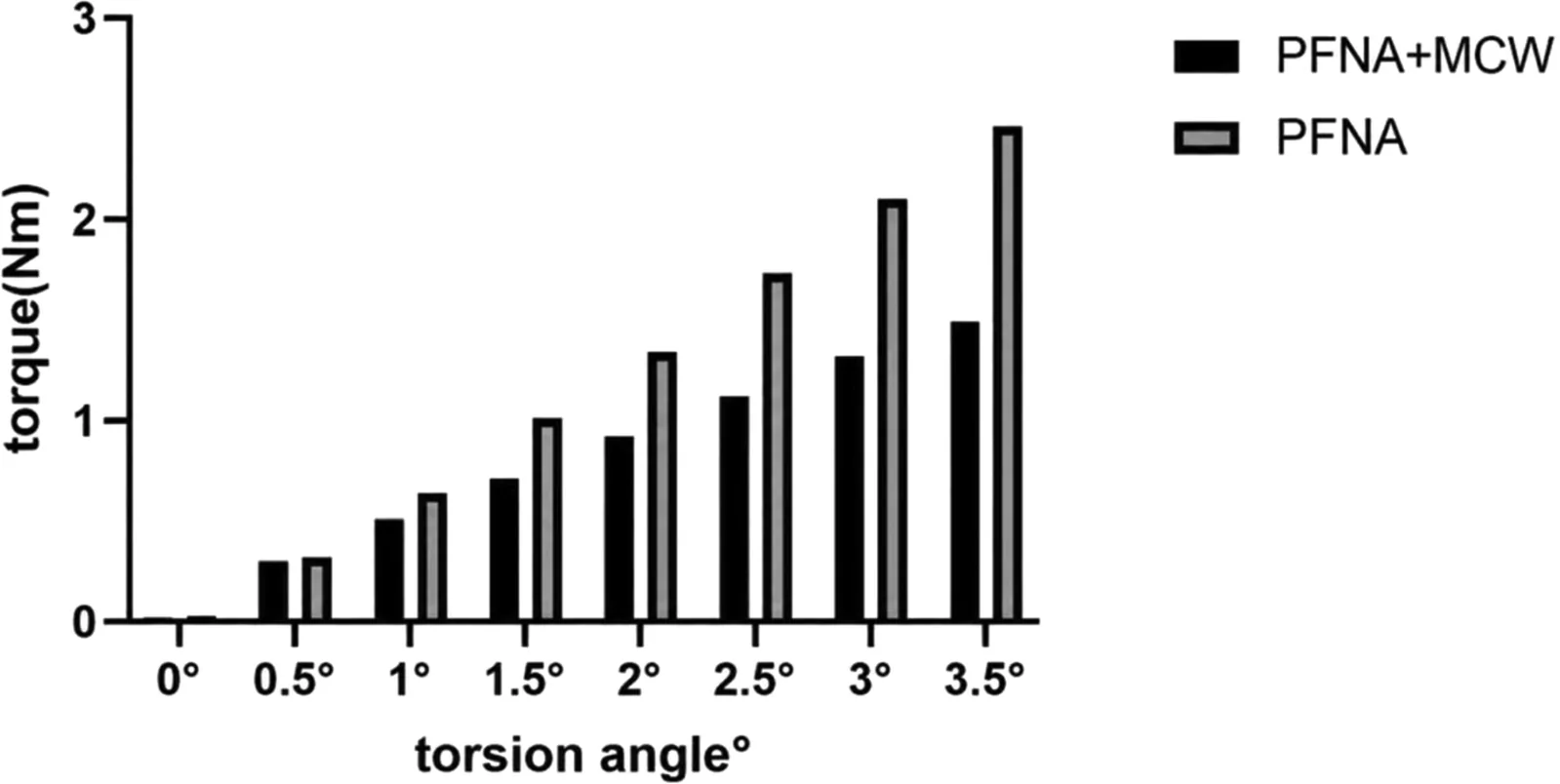
Torque–torsional angle relationship of the 2 fixation constructs. Note: Values are presented for the PFNA + MCW group and the PFNA group at each prescribed torsional angle. MCW = modified candy-package wiring, N·m = newton-meter, PFNA = proximal femoral nail antirotation.

The torsional test results are summarized in Figure 2 and Table 1. The mean torsional angle was 2.09 ± 0.24° in the PFNA + MCW group and 2.90 ± 0.18° in the PFNA group. The torsional angle was significantly lower in the PFNA + MCW group than in the PFNA group (*t* = −5.34, *P* = .0007). The mean torsional stiffness was 0.48 ± 0.0529 N·m/° in the PFNA + MCW group and 0.59 ± 0.0850 N·m/° in the PFNA group. The between-group difference in torsional stiffness was statistically significant (*t* = −2.40, *P* = .045).

**Table 1:** Torsion and axial compression test results.

Outcome	PFNA + modified candy-package wiring (n = 5)	PFNA alone (n = 5)	*t* value	*P* value
Mean torsional angle, °	2.09 ± 0.24	2.90 ± 0.18	−5.34	.0007
Torsional stiffness, N·m/°	0.48 ± 0.0529	0.59 ± 0.0850	−2.40	.045
Maximum failure load, N	571.26 ± 92.75	510.34 ± 76.59	1.13	.291

Note: Data are presented as mean ± standard deviation. *P* values were calculated using two-sided Welch independent-samples *t* tests.

MCW = modified candy-package wiring, PFNA = proximal femoral nail antirotation.

As shown in Figure 2, torque increased progressively with increasing torsional angle in both fixation constructs.

### 3.3. Compression stiffness and failure load

**Figure 3.**
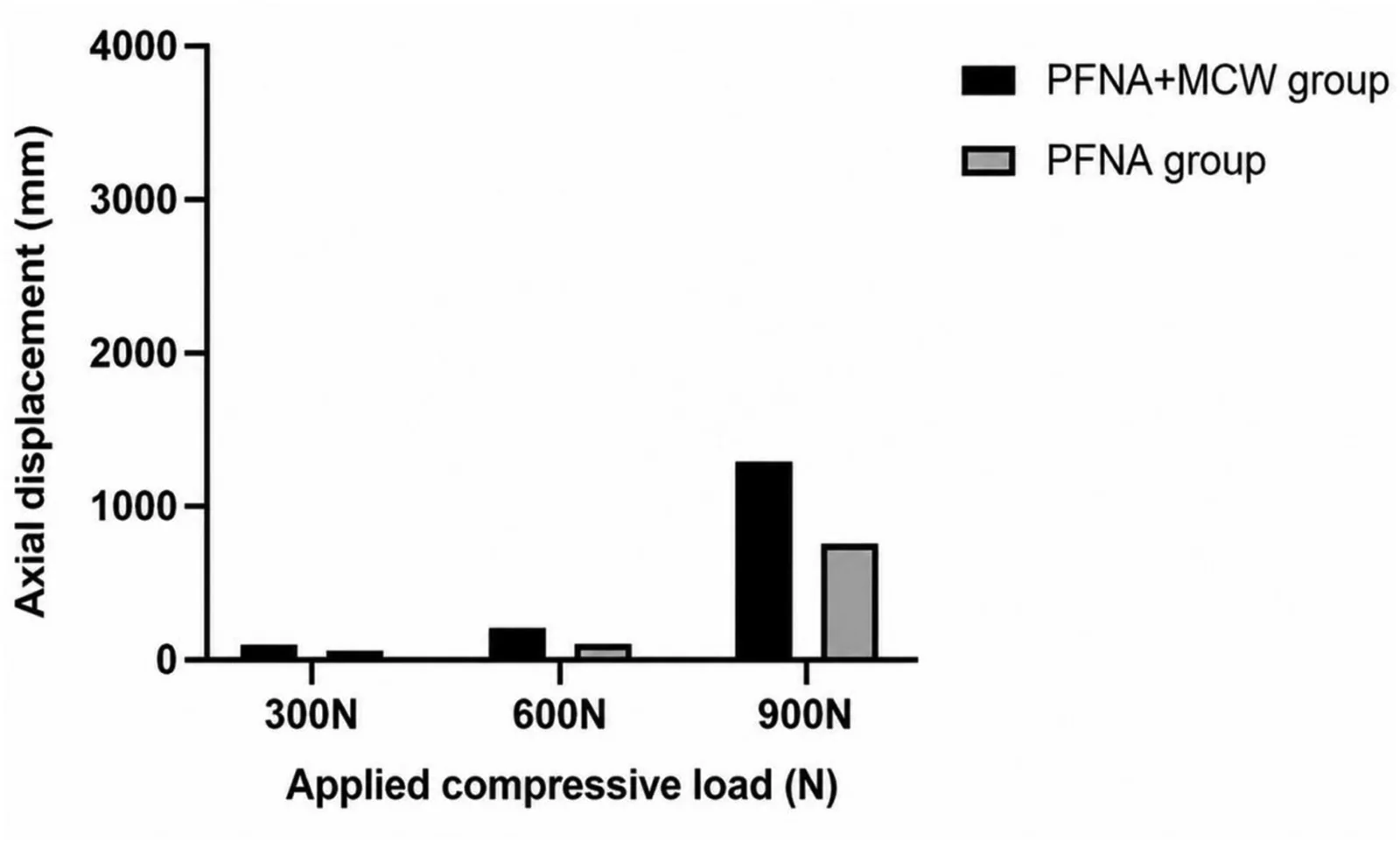
Axial response of the 2 fixation constructs under nominal compressive loads of 300, 600, and 900 N. Note: The vertical-axis outcome should be verified against the original testing-machine export and described consistently as load, displacement, or stiffness before final submission. MCW = modified candy-package wiring, N = newton, PFNA = proximal femoral nail antirotation.

**Figure 4.**
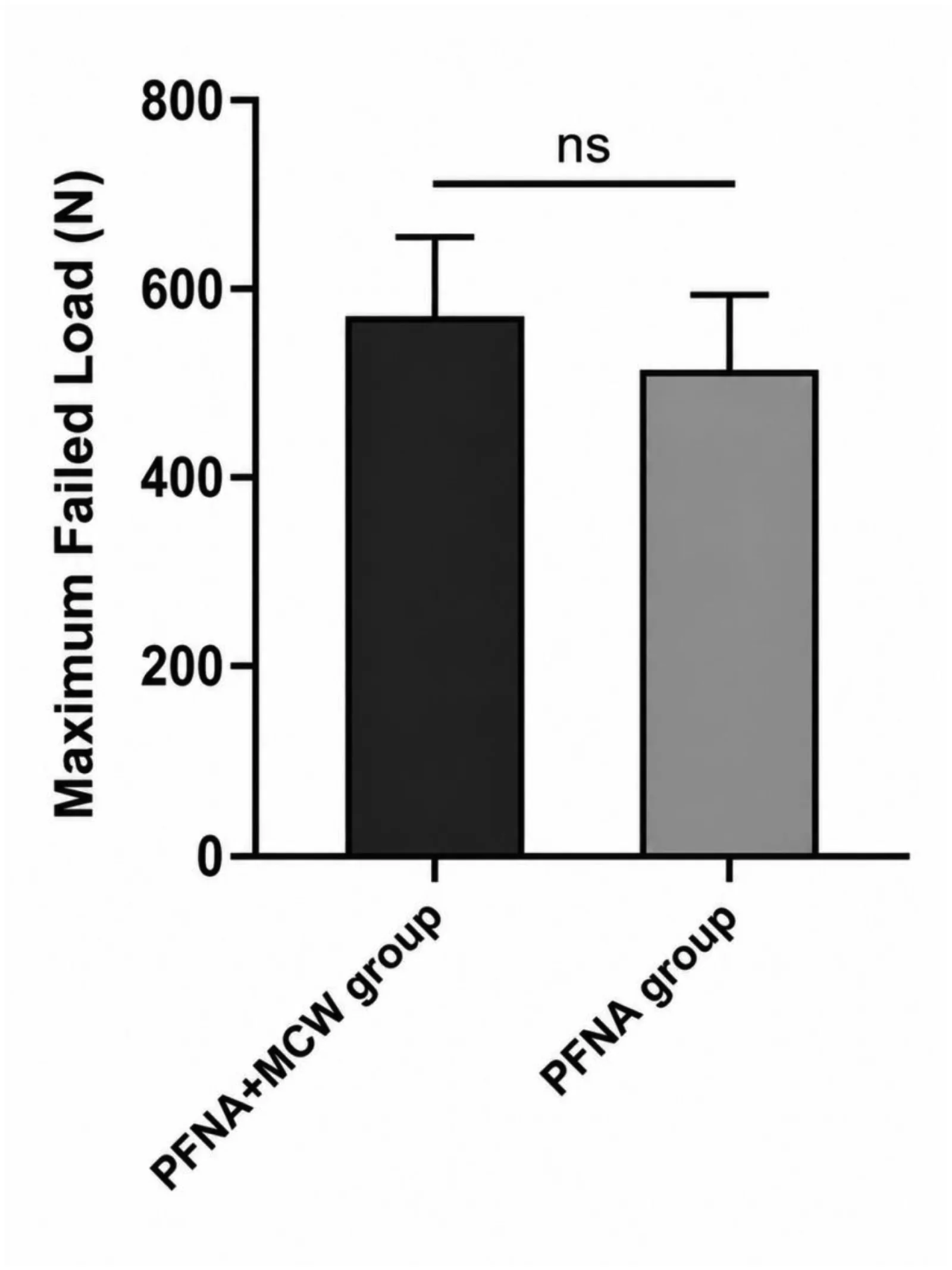
Maximum axial failure load of the 2 fixation constructs. Error bars represent standard deviations. Note: Data are presented as mean ± standard deviation. “ns” indicates no statistically significant between-group difference. MCW = modified candy-package wiring, N = newton, ns = not significant, PFNA = proximal femoral nail antirotation.

The axial displacement responses of the PFNA + MCW and PFNA groups under applied compressive loads of 300, 600, and 900 N are shown in Figure 3. The mean maximum failure load was 571.26 ± 92.75 N in the PFNA + MCW group and 510.34 ± 76.59 N in the PFNA group. Although the mean maximum failure load was numerically higher in the PFNA + MCW group, the between-group difference was not statistically significant (*t* = 1.13, *P* = .291; Table 1 and Fig. 4).

## 4. Discussion

This biomechanical model study compared PFNA combined with modified candy-package wiring (PFNA + MCW) with PFNA alone in an Evans type III intertrochanteric fracture model. PFNA has been widely used for the fixation of unstable intertrochanteric fractures because of its intramedullary mechanical characteristics and relatively favorable load-transfer properties.^[18–20]^ Nevertheless, fixation failure and the need for revision remain important concerns in unstable fracture patterns.^[21,22]^ Against this background, supplementary fixation of unstable fracture fragments may provide additional biomechanical support.

The principal finding of the present study was that the PFNA + MCW group showed a significantly lower torsional angle than the PFNA group (2.09 ± 0.24° vs 2.90 ± 0.24°, *P* = .0007). Under the defined torsional testing conditions, this finding indicates reduced angular displacement of the PFNA + MCW construct and supports improved control of rotational displacement. The modified candy-package wiring technique provides circumferential fixation of the lesser-trochanteric fragment and may therefore help restrict fragment motion in addition to the fixation provided by PFNA.^[21,22]^ However, the present finding should be interpreted specifically as a reduction in rotational displacement under laboratory testing conditions and should not be extrapolated directly to superior clinical outcomes.

A statistically significant difference in torsional stiffness was also observed between the 2 fixation constructs (0.48 ± 0.0529 N·m/° in the PFNA + MCW group vs 0.59 ± 0.0850 N·m/° in the PFNA group, *P* = .045). Importantly, the mean torsional stiffness was lower in the PFNA + MCW group. Therefore, although the difference was statistically significant, it should not be interpreted as evidence that supplementary modified candy-package wiring increased or improved torsional stiffness. Rather, the finding indicates that the 2 constructs exhibited different mechanical responses under torsional loading. In this context, the significantly lower torsional angle provides more direct evidence of reduced rotational displacement in the PFNA + MCW construct.

Previous biomechanical and clinical studies have highlighted several factors that may influence rotational stability and fixation failure in proximal femoral fixation. Excessive or inappropriate mechanical loading may contribute to complications such as the Z-effect and medial migration of cephalomedullary fixation components.^[23,24]^ In addition, disruption or insufficiency of the lateral femoral wall may adversely affect the biomechanical environment of PFNA fixation.^[25]^ These observations provide a biomechanical rationale for exploring supplementary fragment stabilization; however, they do not by themselves establish that PFNA + MCW is biomechanically superior to PFNA alone.

In the axial compression test, the mean maximum failure load was numerically higher in the PFNA + MCW group than in the PFNA group (571.26 ± 92.75 N vs 510.34 ± 76.59 N). However, the between-group difference was not statistically significant (*P* = .291). Accordingly, this finding should be interpreted as a numerical trend rather than evidence of superior axial load-bearing capacity. Previous biomechanical investigations have demonstrated that stress concentration around proximal femoral nail components may contribute to implant deformation or failure under high-load conditions.^[26,27]^ Although supplementary wiring may theoretically alter load distribution by stabilizing fracture fragments, the present data do not demonstrate a statistically significant improvement in maximum failure load.

Schopper et al reported biomechanical benefits of the modified candy-package technique for refixation of the lesser trochanteric fragment.^[14]^ The present study is generally consistent with the rationale that supplementary fixation may influence construct stability, particularly through improved control of fragment displacement. Nevertheless, the current findings should not be interpreted as demonstrating superiority across all biomechanical parameters. Specifically, although torsional angle was significantly lower in the PFNA + MCW group and torsional stiffness differed significantly between the groups, the stiffness value itself was lower in the PFNA + MCW group, and the difference in maximum failure load did not reach statistical significance.

## 5. Limitations

This study has several important limitations. First, the sample size was only 5 models per group, resulting in low statistical power, wide confidence intervals, and unstable estimates of variance and normality. A non-significant result must not be interpreted as equivalence. Second, synthetic femoral models cannot reproduce the biological heterogeneity, bone quality, soft-tissue constraints, healing response, or cyclic loading encountered clinically, limiting generalizability. Third, only the final record after conditioning was analyzed; repeatability statistics and within-specimen coefficients of variation were not available. Fourth, fatigue testing and cyclic failure analysis were not performed. Future biomechanical studies should include larger sample sizes, report effect sizes and confidence intervals, quantify repeatability between cycles, and incorporate cyclic loading and failure-mode analyses. Clinical studies are also needed before the findings can be translated into recommendations for patient care.

## 6. Conclusion

In this biomechanical model study, PFNA combined with modified candy-package wiring resulted in a significantly lower torsional angle than PFNA alone, indicating reduced rotational displacement under the defined testing conditions. A statistically significant difference in torsional stiffness was also observed between the 2 fixation constructs, although the lower stiffness value in the PFNA + MCW group should not be interpreted as superior torsional stiffness. The PFNA + MCW group showed a numerically higher maximum failure load, but the difference was not statistically significant. Given the small sample size, these findings should be interpreted as preliminary biomechanical evidence and require confirmation in larger biomechanical and clinical studies.

## Acknowledgments

The authors would like to express their gratitude to all the participants and staff who were involved in this study.

## Author contributions

**Conceptualization:** Qiong Chen, Xiaan Chen, Suqi Wu, Hansong Wu, Rongrong Chi, Xiangu Li, Jiaqi Wang.

**Data curation:** Qiong Chen, Xiaan Chen, Suqi Wu, Hansong Wu, Rongrong Chi, Xiangu Li, Jiaqi Wang.

**Formal analysis:** Qiong Chen, Xiaan Chen, Suqi Wu, Hansong Wu, Rongrong Chi, Xiangu Li, Jiaqi Wang.

**Funding acquisition:** Qiong Chen, Xiaan Chen, Jiaqi Wang.

**Investigation:** Qiong Chen, Jiaqi Wang.

**Writing – original draft:** Jiaqi Wang.

**Writing – review & editing:** Jiaqi Wang.
